# Exome-based Variant Detection in Core Promoters

**DOI:** 10.1038/srep30716

**Published:** 2016-07-28

**Authors:** Yeong C. Kim, Jian Cui, Jiangtao Luo, Fengxia Xiao, Bradley Downs, San Ming Wang

**Affiliations:** 1 Department of Genetics, Cell Biology and Anatomy, College of Medicine, Omaha, NE 68198, USA; 2 Department of Biostatistics, College of Public Health, University of Nebraska Medical Center, Omaha, NE 68198, USA.

## Abstract

Core promoter controls the initiation of transcription. Core promoter sequence change can disrupt transcriptional regulation, lead to impairment of gene expression and ultimately diseases. Therefore, comprehensive characterization of core promoters is essential to understand normal and abnormal gene expression in biomedical studies. Here we report the development of EVDC (Exome-based Variant Detection in Core promoters) method for genome-scale analysis of core-promoter sequence variation. This method is based on the fact that exome sequences contain the sequences not only from coding exons but also from non-coding region including core promoters generated by random fragmentation in exome sequencing process. Using exome data from three cell types of CD4+ T cells, CD19+ B cells and neutrophils of a single individual, we characterized the features of core promoter-mapped exome sequences, and analysed core-promoter variation in this individual genome. We also compared the core promoters between YRI (Yoruba in Ibadan, Nigeria) and the CEU (Utah residents of European decedent) populations using the exome data generated by the 1000 Genome project, and observed much higher variation in YRI population than in CEU population. Our study demonstrates that the EVDC method provides a simple but powerful means for genome-wile *de novo* characterization of core promoter sequence variation.

Transcription initiation is regulated through highly specific spatial interaction between cis- and trans-elements in the core promoter^1^,^2^,^3^. Alterations in core promoter cis-sequences can affect cis-trans interaction, disturb formation of the initiation complex machinery, resulting in altered gene expression and diseases including cancer^4^,^5^,^6^,^7^,^8^,^9^,^10^,^11^. A typical eukaryotic core promoter contains multiple conserved cis-regulatory elements located about 50-bases upstream and downstream of the transcriptional start site (TSS)^1^,^2^, including the TFIIB recognition element (BRE), TATA box, Initiator element (INR), downstream promoter element (DPE), and other transcription factor binding sites (TFBS).

Core promoters in several model species have been extensively annotated through many studies, and the information provides rich resources to study regulation of transcription initiation^1^,^2^,^3^. Many biological and medical studies require gaining genomewide, *de novo* information to study the regulation of transcription initiation under specific physiological and pathological conditions. However, current methodologies have limited power to accomplish this task. For example, ChIP-sequencing (e.g., ChIP-exo) can only analyse a given type of trans-element binding sites in one assay^12^; promoter-array^13^ has low specificity to provide gene-specific probes due to the highly conserved core promoter sequences among different genes, and has inherited deficiency to detect altered core-promoters as the probes designed for the array are based on normal reference genome sequences; Cap Analysis Gene Expression (CAGE) can locate promoters precisely but it does not analyse core promoter contents^14^; and whole-genome sequencing is not cost-effective as core promoters account for only a tiny portion of the entire genome.

Here, we describe the development of the EVDC (Exome-based Variant Detection in Core-promoters) method for core promoter analysis. Exome sequencing was designed to analyse the coding-exons in a genome^15^. However, it has been observed that over half of exome sequences are routinely originated outside of coding exons^16^,^17^. This is caused by the random fragmentation of genomic DNA used in exome library preparation^18^, which generates both coding-exon templates and coding-exon connected non-coding templates including core promoters. Isolation of DNA templates through hybridization with exon-specific probes collects not only coding-exon templates but also coding-exon connected core promoter templates. Sequencing the isolated DNA templates will generate the sequences derived from core promoters (Fig. 1).

## Results

### Characterization of promoter-mapped exome sequences

**Figure 2 f2:**
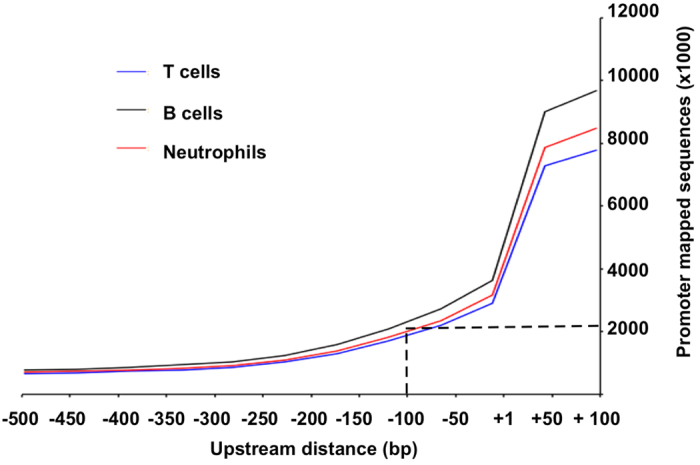
Distribution of promoter-mapped exome sequences. It shows that the promoter-mapped sequences provide sufficient coverage in core-promoter region between −100 to +100. The same patterns appeared in all three data set of T cells, B cells and neutrophils.

**Figure 3 f3:**
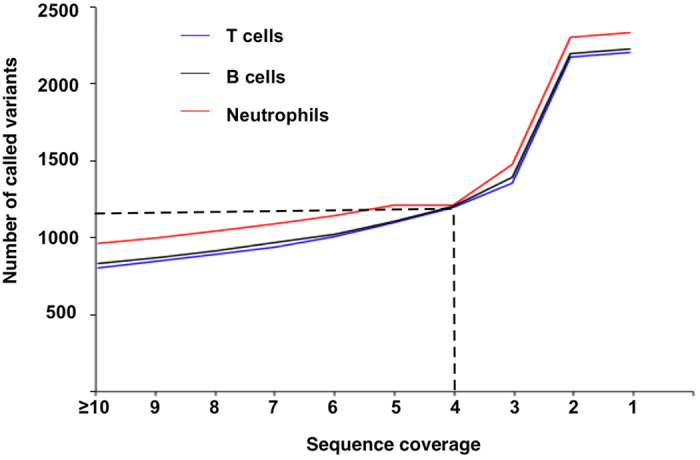
Relationship between sequence coverage and variant call. It shows the steady increases of variants called from coverage > =10 to 3.

We analysed the relationship between core-promoters and exome sequences. As a model for the study, we used three sets of exome sequences generated independently from human CD4+ T cells, CD19+ B cells and neutrophils of a healthy Caucasian male^19^. The use of these three sets of exome sequence data from the same individual allowed us to measure the reproducibility of results. We performed the following analyses:

1. Portion of exome sequences from promoter region. We mapped the three sets of exome sequences to the human reference genome (hg19) to determine the origins of the exome sequences (Table 1). Many genes have multiple TSS sites, and the region downstream TSS sites is also involved in transcription initiation^20^. Therefore, we included the region between −500 to +100 for the measure. The results showed that on average, an exome data set contained over six million bases (1.63% of the total exome data) originated from promoter region between −500 to +100 (Table 1);
2. Distribution of promoter-mapped exome sequences. Between −500 to +100, the promoter-mapped sequences had the highest abundance at +100 and then decreased towards more upstream. At −100 upstream TSS, there were on average around 2 million bases from promoter-mapped exome sequences in each exome data set (Fig. 2);
3. Number of promoters detected by exome sequences. The highest number of promoters detected was at +100, covering 85.1% promoters for the 20,794 genes targeted by the exome kit. The rates decreased towards upstream. At −100 upstream TSS, on average 78.7% of the promoters were detected in each exome data set (Table 2);
4. Variant call and sequence coverage. We called the variants from promoter-mapped sequences from high coverage till single sequence coverage. The number of called variants increased smoothly from >=10 to 3 sequences. Thereafter, the number of called variants increased substantially. However, the reliability of such increased variants is questionable as they could be originated from sequence errors in the 2 to 1 mapped sequences (Fig. 3).

[Table t1][Table t2]

Based on these results, we conclude that the promoter-mapped exome sequences are most suitable to analyze core promoters between −100 to +100, and a minimum of four-sequence coverage should ensure the reliability of the variants called from the promoter-mapped sequences.[Fig f2][Fig f3]

### Variants called from the core promoters in the individual genome

**Figure 4:**
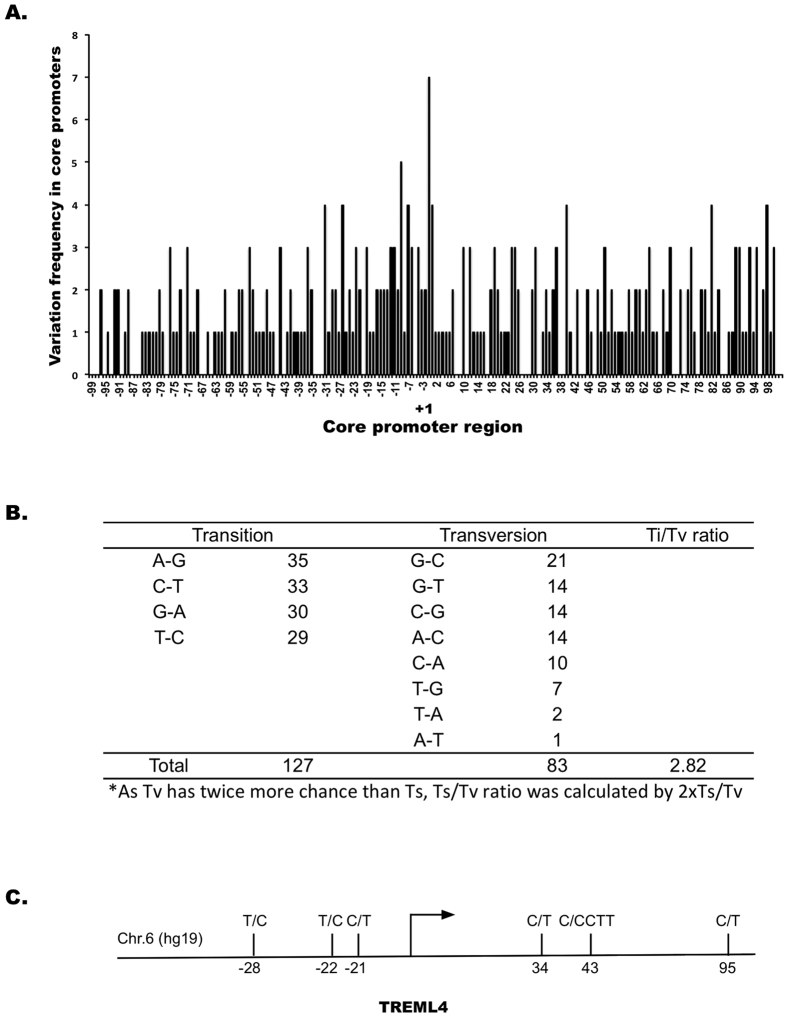
The 291 variants identified in core promoters of the exome-sequenced individual genome. (**A**) Distribution of the 291 variants between +100 and −100 at the frequencies between 1 to 7. (**B**) Types of variants and Ts/Tv ratio. Of the 291 variants, A to G transition had the highest number of 35 times, whereas A to T transversion had the lowest number of only 1 time. (**C**) The presence of six transition variants between C and T in core promoter of *TREML4* between −28 to 95.

**Figure 5 f5:**
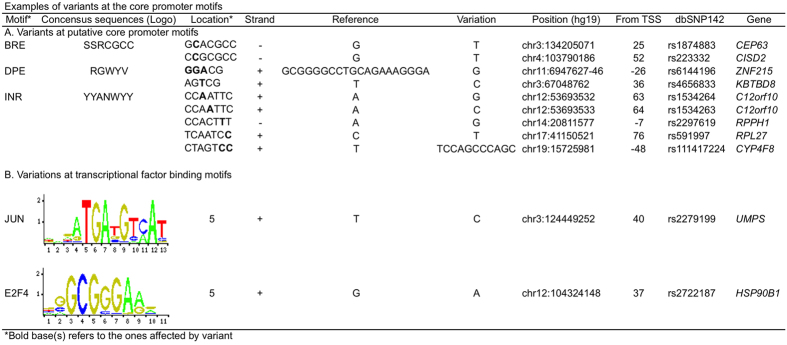
Variant-affected motifs in the exome-sequenced individual genome. (**A**) Variant-affected core promoter motifs; (**B**) Variant-affected transcriptional factor binding motifs. Note that the motif bases were based the top strand whereas the variant bases were based on the actual strand depending on its orientation.

Using the conditions set above, we analyzed the core promoter variation in this individual genome using hg19 as the reference. We identified 291 variants distributed at different frequencies within the core-promoter region (Fig. 4A, Supplementary Table 1). We characterized these variants:

1. Reliability of called variants. Of the 291 called variants, 288 (98.9%) already exist in dbSNP and 253 (86%) had high Minor Allele Frequency (>0.05). Sanger sequencing validated 90% (45 of 50) of the randomly selected variants called from the ≥four mapped sequences (Supplementary Table 2). These results highlighted that the variants called on the conditions were highly reliable.
2. Features of the called variants. The 291 variants consisted of 81 indels ranging from 2 to 23 bases, and 210 SNPs. No obvious base preference was observed for the indel sites (35, 33, 30 and 29 in the order of A, C, G and T), but substantial differences existed for the SNP sites, with the highest A to G transition (Ts) for 35 times, and the lowest A to T transversion (Tv) for only once. Ts/Tv ratio was 2.82, which is higher than the 2–2.1 across the entire human genome (Fig. 4B).
3. Genes with variable core promoters. The 291 variants were distributed in the core promoters of 241 genes. The core-promoters in multiple genes, including *CRIP1, HLA-F-AS1, ISG15, OR1N2*, and *TREML4*, were highly variable with three to six variants. For example, there were six variants located at −28, −22, −21, 34, 43, and 95 across core-promoter of *TREML4* (triggering receptor expressed on myeloid cells-like 4), a gene involved in antigen process in myeloid cells and T cells^21^ (Fig. 4C).
4. Core promoter motifs with variants. Searching for the variants located in core promoter motifs of BRE, TATA box, INR and DPE identified 3 variants in the BRE of three genes, 15 variants in the DPE of 13 genes, and five variants in the INR of three genes (Supplementary Table 1). For example, two variants of A to G and A to C were at the third and fourth positions of a putative INR motif (CCAATTC) in the core promoter of *C12orf10* (Fig. 5). We did not find any variants in TATA boxes, suggesting that TATA box sequences in this individual genome are stable. Searched for variants located in conventional transcriptional factor binding motifs (TFBS) within core promoters identified the variants located in two TFBS of two genes, including a variant in JUN binding motif at +40 of the *UMPS* core promoter and a variant in E2F4 binding motif at +37 of the *HSP90B1* core promoter. Each of the variants was located at the intolerant position of its TFBS with the Position Frequency Matrix (PFM) index of 2 (Fig. 5).

### Core-promoter variation between Caucasian and African genomes

[Fig f5]It is well known that African genomes are more divergent than those of other ethnicities are^22^,^23^. Using the EVDC method, we compared the similarity and difference of core promoters between two ethnical groups, the YRI (Yoruba in Ibadan, Nigeria) and the CEU (Utah residents of European decedent), using 27 exome data sets from each group collected by the 1000 Genome Project^24^ for the analysis (Supplementary Table 3). The results showed that

**Figure 6 f6:**
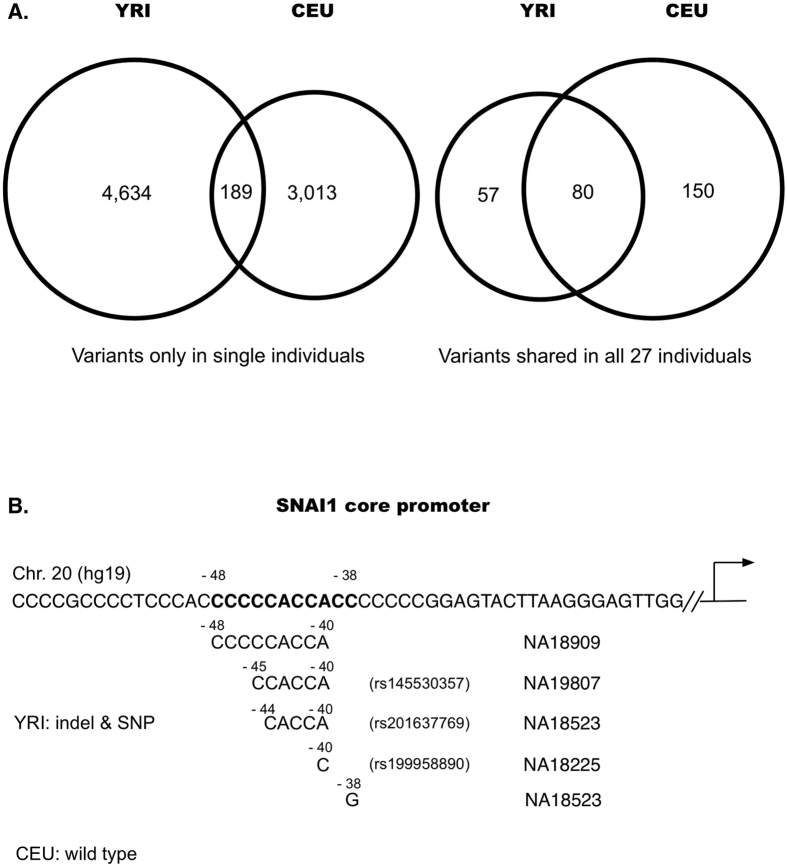
Comparison of variants between YRI and CEU populations. (**A**) Comparisons of the variants present only in single individuals of YRI and CEU populations, and the variants shared in all 27 individuals of YRI and CEU populations. (**B**) Comparison of SNAI1 core promoter between YRI and CEU populations. In the individualized variants, five variants of 3 deletions and two SNPs, of which three are known variants in dbSNP, were present in *SNAI1* core promoter between −48 to −38 in four YRI individuals, the SNP at −38 (C > G) converted CCACCCC into a BRE site CCACGCC, whereas all CEU individuals remained the same as in the reference genome sequences (hg19).

1. Core promoters were more variable in YRI than in CEU. There were a total of 14,372 variants in YRI comparing to 11,380 in CEU (p = 1.51E^−30^), and 4,823 variants in single individuals in YRI group comparing to 3,202 in CEU (p = 9.71 × 10^−21^). Variants shared in multiple individuals within each group were more common in CEU than in YRI, as exemplified by the 230 variants common in all 27 CEU individuals comparing to 137 in YRI (p = 6.97E10^−13^) (Table 3, Supplementary Tables 4A–C and 5A–C). The fluctuated P-values among the variants between YRI and CEU subgroups were likely caused by both technical and biological factors, such as the limited sample size used in the study and non-random distribution of variants in the core-promoters;
2. Features of the variants in YRI and CEU. There were 19 indels in the variants shared within YRI and CEU groups, but the numbers increased to 816 in the variants only present in single individuals in YRI and CEU groups. In the shared variants, CEU had more Indels than in YRI (12 to 3); but in the individualized variants, YRI had more than in CEU (475 to 320). Similar patterns were present for SNPs: there were 268 SNPs in the variants shared within YRI and CEU groups but the numbers increased to 7,021 in the variants only present in single individuals in YRI and CEU groups, and the number in individualized YRI group was much larger than in CEU group (4,159 to 2,693). The highest SNP change was G to A in individualized variants in YRI (940) and the lowest SNP change was T to A in individualized variants shared between YRI and CEU groups (1 only). Ts/Tv ratios were all over 3 in each subgroup except 2.45 in the shared variants between the variants not shared within YRI and CEU groups (Table 4).
3. Individualized variants were mostly known variants. For the 4,823 YRI and 3,202 CEU individualized variants, 75% in YRI and 74.7% in CEU were present in dbSNP (Supplementary Table 4A–C); for the variants common in all 27 cases in each group, 100% in YRI, 100% in CEU, and 98.8% of the 80 shared variants were present in dbSNP (Supplementary Table 5A–C). This information further supports the reliability of the variants called from YRI and CEU groups.
4. Individualized variants were mostly not shared between YRI and CEU groups. Of the 3,202 CEU and 4,823 YRI individualized variants, only 189 (4% in YRI and 6% in CEU) were shared between the two groups. In contrast, of the 137 and 230 variants common in all 27 cases of YRI and CEU groups, 80 variants (58% YRI and 35% CEU) were shared between the two groups (Fig. 6A);
5. More genes in YRI contained variable core promoters than these in CEU. In the individualized variants, 3,741 genes in YRI had their core promoters affected by 4,823 variants but only 2,557 genes in CEU affected by 3,202 variants. Taking SNAI1 as an example: SNAI1 is a zinc finger transcriptional repressor related with a parasitic infectious disease Dracunculiasis. Together with histone demethylase KDM1A, SNAI1 decreases dimethylated H3K4 levels to repress transcription involved in mesoderm formation during embryonic development. In the individualized variants in YRI, five variants of 3 deletions and two SNPs were present in *SNAI1* core promoter between −48 to −38 in four individuals, the SNP at −38 (C > G) converted the wild type sequence CCACCCC into a BRE site CCACGCC. In comparison, *SNAI1* promoter in CEU group maintained wild type sequences (Fig. 6B).
6. Core promoter motifs in YRI were more affected than these in CEU. Of the variants common in 27 cases, 17 motifs were affected by the variants in YRI only compared to 8 only in CEU; of the individualized variants, 402 motifs were affected by the variants in YRI only compared to 259 in CEU only. The most affected motif was DPE in both groups. Only one and three variants in shared and individualized YRI variants were in TATA box but none in CEU group, indicating that TATA box sequences in these two ethnical groups are stable (Table 5). Searching for the variants affecting TFBS didn’t find any in the shared variants in either groups except two individualized variants in YRI group in ZBTB33 and MYC sites of *MAPK15* and *UQCRBP1*, and two in CEU group in Egr1 and CTCF sites of *RICTOR* and *RMRP* (data not shown).

Data from these analyses revealed that the core promoters in YRI population are more variable than in CEU population.[Fig f6][Table t3][Table t4][Table t5]

## Discussion

The unique features of the EVDC method include: (1) It provides *de novo* information without the need for *a priori* knowledge of core promoter sequences. As the promoter sequences are collected indirectly through probes targeting coding exons, they preserve the native information of the core promoter sequences; (2) It detects the variants in core promoters at genome level. This is achieved by taking the advantage of exome sequences targeting the entire coding genes; (3) It extends the value of exome sequences from the coding region to the core-promoter region; (4) It is cost-effective.

Around 80% of gene core promoters were confidently detected by our method but 20% of promoters were missed (Table 2). A part of the missed promoters was in fact mapped by exome sequences but at lower coverage. Although variants from these promoters could be called, these called variants are less reliable and distributed in more random manner, which is troublesome, particularly when comparing the variants between different genomes. Other cause for the missed promoters can be attributed by the lack of coding-exon connected core promoter templates. We quantified these missed promoters in the 3 exome data sets from the donor. The results showed that 12.4% were missed in all three samples and the remaining had lower sequence coverage. Improvement of designing exon probes closer to the 5′ end of 5′-UTR, generating longer fragments during random fragmentation should increase the rate of core promoter detection.

Sequence length can influence the promoter region covered by exome sequences. In our study, we used paired-end read at 2 × 100 by Illumina sequencer HiSeq2000. All Illumina sequencers use bridge amplification-based cluster generation and sequencing by synthesis. The nature of these methods determines that the rate of artificial bases will significantly increase after 100 bases^22^. Although new Illumina sequencers can provide longer reads up to 300 bp, causing needs to be taken that increased sequencing errors towards the longer sequence ends can be problematic for variation analysis in core-promoter region.

Our data show that over 80% of exome sequences were from non-coding regions. This rate is higher than previously reported 50–65%^16^,^17^,^25^. We quantified the rate in the 27 CEU exome data sets from the 1000 genome project (Supplementary Table 3), which were generated by using early-generation exome kits. The results show that 60% of the sequences were from non-coding regions. This difference can be due to the different exome kits used in the studies. Exome kits are under constant evolution. The early exome data were collected by using the first-generation exome kits, which mainly targeted the coding exons^24^. Later on, all exome kits have doubled their targeted regions by inclusion of 5′ UTR, intron-exon boundary, 3′ UTR, and non-coding genes etc. As such, the proportion of the exome sequences from the non-coding region has been substantially increased over these generated by the early exome kits. This feature increases the value of using exome sequences for non-coding sequence analysis.

Many variant-affected core promoter motifs were not located at the conventionally defined positions in core promoters. For example, none of the 15 variant-affected DPE motifs identified in the individual genome was located within the DPE position defined as 27–33 bases downstream of TSS site^1^,^2^. A possibility is that certain variant-affected core promoter motifs at the non-conventional positions could be related to one of the multiple TSS, a common phenomenon existing in many genes^26^. Another possibility can be related with the limited length of the consensus motif sequences. BPE and INR motifs have seven bases but DPE has only five bases, which makes variant-mapped DPE motif less specific or even simply hit by random chance^27^.

We compared the sensitivity of exome sequences and whole genome sequences for core-promoter variant detection by using the exome and whole genome data of the same individual from 1000 Genome project^28^. Comparing the variants between the data sets shows that 79% of exome detected variants were also detected by whole genome sequences, but the later detected 1.4 times more variants than exome sequences did; furthermore, a portion of variants was only detected by each method (Supplementary Figure 1). While whole genome sequences can detect more variants than exome sequences, the sequencing cost is an important factor to consider. Current cost of whole genome sequencing is about 3–5 times higher than that of exome sequencing. We consider that at current art of sequencing technologies, exome sequencing is a more cost-effective solution than whole genome sequencing for core promoter analysis.

Large quantities of publically available exome data have been collected by various genomics projects, such as the Exon Variant Server Project^29^, the 1000 Genome Project^30^, the Cancer Genome Atlas Project^31^, and the Exome Aggregation Consortium^32^. These exome data have been used primarily for coding-exon analyses. Applying the EVDC method to mine these rich exome resources should lead to a comprehensive characterization of core-promoters in humans and other species for better understanding of transcriptional initiation regulation.

## Materials and Methods

### Samples used for the study

CD4+ T cells, CD19+ B cells and neutrophils from venous blood of a healthy Caucasian male donor were purchased from AllCells Company (AllCells, Alameda, California). The use of the cells for research purpose has AllCells company IRB approval (Biomed IRB). The donor signed the written consent form, which is archived with their medical records. All experiments were performed in accordance with relevant guidelines and regulations. According to US Federal Regulations, 45 CFR Part 46.101(b)(4)–Protection of Human Subjects, using this type of human cells for research is exempted from the requirement for IRB review.

### Exome sequences

Exome sequences were generated as reported^19^. Briefly, genomic DNA from three purified cell types of CD4+ T cells, CD19+ B cells and neutrophils of the individual was extracted using a FlexiGene DNA kit (Qiagen). An exome library was constructed for each DNA sample by random fragmentation using the Covaris II system (Covaris Inc. Massachusetts, USA), and by isolation of exome templates using the TruSeq Exome Enrichment Kit (Illumina^®^) following the manufacturer’s protocols. Exome sequences were collected by using a HiSeq 2000 sequencer (paired-end 2 × 100). Exome sequence data were deposited in the National Center for Biotechnology Information (NCBI) with accession number SRR933549. Exome data from the 1000 genome project were downloaded from 1000 Genomes FTP site^24^.

### Exome data mapping and variant call from promoter-mapped exome sequences

Exome sequences were mapped to the human genome reference sequence hg19 by Bowtie2 using the default parameters in paired mode^33^. The resulting SAM files were converted to BAM files and sorted. Duplicates were removed using the Picard program. The mapped reads were processed using Genome Analysis Toolkit^34^ with RealignerTargetCreator and IndelRealigner for local realignment. BaseRecalibrator was used for base quality score recalibration. GATK HaplotypeCaller was used for variant calls, followed by variant recalibration with VariantRecalibrator. The called variants were annotated with ANNOVAR^35^ using RefSeq, dbSNP144 and 1000 Genomes databases. Promoter regions between −500~+100 of the exome kit-targeted 20,794 genes were extracted from hg19. A minimum of four promoter-mapped sequences was set for variant calling. For overlapped genes, the first gene was used for the mapping analysis.

For variants located in core promoter motifs, the following consensus sequences were used as the references^1^,^2^: SSRCGCC (BRE), TATAWAAR (TATA box), YYANWYY (INR), and RGWYV (DPE). For the variants located at transcription factor binding motif, the motifs listed at JASPAR CORE database for human were used as the reference^36^.

## Additional Information

**How to cite this article**: Kim, Y. C. *et al*. Exome-based Variant Detection in Core Promoters. *Sci. Rep.* **6**, 30716; doi: 10.1038/srep30716 (2016).

## Supplementary Material

Supplementary Table 1

Supplementary Table 2

Supplementary Table 3

Supplementary Table 4

Supplementary Table 5

Supplementary Figure 1

## Figures and Tables

**Figure 1:**
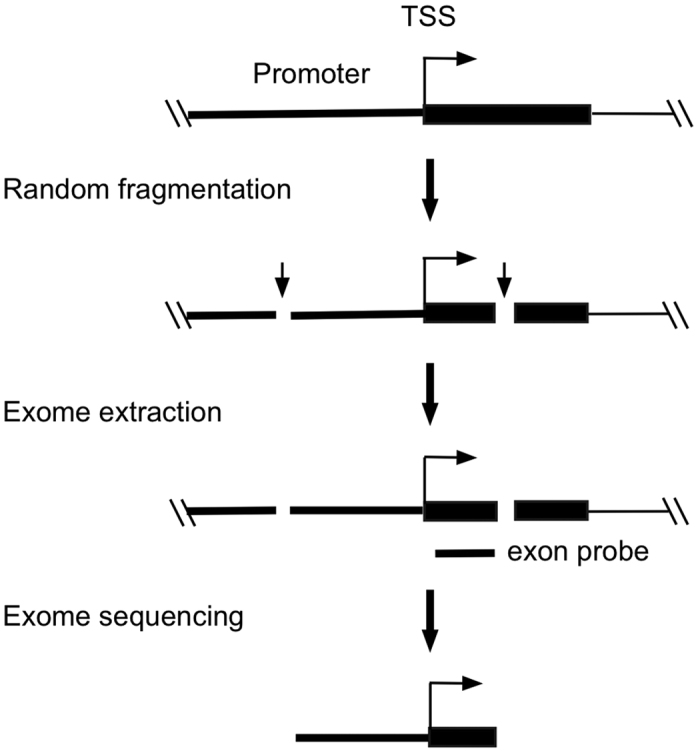
Scheme of the EVDC method. It shows that random fragmentation of genomic DNA generates the DNA templates connecting core-promoters and coding exons. Exon-targeting probes will isolate such templates. Sequencing the isolated templates will result in the sequences originated from core-promoters.

**Table 1 t1:** Origins of exome sequences.

Region	Bases in hg19	Bases in exome sequences (%)*			
		CD4+ T cells	CD19+ B cells	Neutrophils	Average (%)
Promoter (−500 to +100)**	12,836,200 (0.40)	6,703,112 (1.40)	6,778,727 (1.94)	6,733,285 (1.54)	1.63
Exon (−100)	59,850,595 (1.91)	57,657,126 (12.06)	57,866,861 (16.58)	57,768,091 (13.23)	13.96
Intron	1,018,096,195 (32.45)	197,986,090 (41.43)	154,196,338 (44.17)	184,099,512 (42.17)	42.59
Intergenic	2,046,361,703 (65.23)	215,552,155 (45.10)	130,266,507 (37.31)	187,981,809 (43.06)	41.82
Total	3,137,144,693 (100)	477,898,483 (100)	349,108,433 (100)	436,582,697 (100)	100.0

^*^Rate (%) was calculated as: bases in a region/total bases in corresponding items.

^**^100 bp added from exon part.

**Table 2 t2:** Number of promoters mapped by exome sequences.

Promoter region	Promoter detected			
	CD4+ T cells	CD19+ B cells	Neutrophils	Average %
Total gene targeted*	20,794 (100)	20,794 (100)	20,794 (100)	100
Promoter detected				
Downstream TSS				
100 to 51	17,357 (83.5)	18,002 (86.6)	17,719 (85.2)	85.1
50 to 1	17,047 (82.0)	17,678 (85.0)	17,370 (83.5)	83.5
Upstream TSS				
1 to 50	16,761 (80.6)	17,379 (83.6)	17,022 (81.9)	82.0
51–100	16,072 (77.3)	16,650 (80.1)	16,347 (78.6)	78.7
101–150	15,065 (72.4)	15,480 (74.4)	15,287 (73.5)	73.5
151–200	14,128 (67.9)	14,384 (69.2)	14,918 (71.7)	69.6
201–250	13,047 (62.7)	13,284 (63.9)	13,131 (63.1)	63.3
251–300	11,940 (57.4)	11,929 (57.4)	11,905 (57.3)	57.3
301–350	10,461 (50.3)	10,223 (49.2)	10,372 (49.9)	49.8
351–400	8,841 (42.5)	8,364 (40.2)	8,610 (41.4)	41.4
401–450	7,478 (36.0)	6,667 (32.1)	7,112 (34.2)	34.1
451–500	6,473 (31.1)	5,539 (26.6)	6,196 (29.8)	29.2

^*^Total number of genes targeted by Illumina TruSeq exome enrichment kit.

**Table 3 t3:** Differences of core promoter variants between YRI and CEU populations.

Number of samples sharing the same variant	Number of variants		P value (χ^2^ test)
	YRI	CEU	
1	4,823	3,202	3.99E-21
2	2,195	1,585	0.002
3	1,381	1,075	0.659
4	947	807	0.112
5	726	668	0.004
6	564	507	0.034
7	441	421	0.005
8	367	343	0.025
9	339	303	0.120
10	255	253	0.010
11	213	218	0.007
12	180	182	0.019
13	203	185	0.163
14	140	165	4.57E-04
15	181	143	0.961
16	122	147	0.001
17	148	118	0.955
18	129	92	0.441
19	137	101	0.584
20	113	104	0.266
21	104	77	0.654
22	97	101	0.052
23	71	82	0.019
24	113	98	0.508
25	102	86	0.667
26	144	87	0.045
27	137	230	6.97E-13
Total	14,372	11,380	1.51E-30

**Table 4 t4:** Ts/Tv ratio for the variants in YRI and CEU populations.

Change	Variants shared within group			Variants not shared within group		
	YRI only	CEU only	Shared	YRI only	CEU only	Shared
Indel	3	12	4	475	320	22
SNPs						
Transition						
A-G	11	29	21	415	293	16
C-T	3	9	8	899	578	25
G-A	7	18	6	940	556	33
T-C	12	30	11	396	265	19
subtotal	33 (61)	86 (62)	46 (61)	2,650 (64)	1,692 (63)	93 (55)
Transversion						
A-C	3	11	6	127	106	6
A-T	2	3	6	96	55	5
C-A		7	2	245	171	10
C-G	5	7	2	286	186	20
G-C	4	9	3	264	164	14
G-T	2	2	4	268	164	10
T-A	2	6	3	79	65	1
T-G	3	7	4	144	90	10
Subtotal	21 (39)	52 (38)	30 (39)	1,509 (36)	1,001 (37)	76 (45)
Total	54 (100)	138 (100)	76 (100)	4,159 (100)	2,693 (100)	169 (100)
Ts/Tv ratio*	3.14	3.31	3.07	3.51	3.38	2.45

^*^Ts/Tv ratio was calculated by 2xTs/Tv.

**Table 5 t5:** Variants distributed in putative core promoter motifs.

Item	Group		
	YRI	CEU	In both
Variants common in 27 cases			
Putative motif affected	17	8	8
BRE	0	0	0
TATA box	1	0	0
INR	6	0	6
DPE	10	8	2
Variants individualized			
Putative motif affected	402	259	15
BRE	40	19	1
TATA box	3	0	0
INR	141	91	8
DPE	218	149	6
