# Supplementary material for: Exome-based Variant Detection in Core Promoters
Source: Sci Rep. 2016 Jul 28;6:30716. doi: 10.1038/srep30716 (PMC4964598; doi:10.1038/srep30716)
Supplement: Supplementary Figure 1 [file srep30716-s6.doc]

**Exome-based Variant Detection in Core Promoters**

Yeong C. Kim, Jian Cui, Jiangtao Luo, Fengxia Xiao, Bradley Downs, San Ming Wang
